# Balancing Objectivity and Welfare: Physiological and Behavioural Responses of Guide Dogs During an Independent Certification Protocol

**DOI:** 10.3390/ani15131896

**Published:** 2025-06-26

**Authors:** Viola Faerber-Morak, Lisa-Maria Glenk, Karl Weissenbacher, Annika Bremhorst

**Affiliations:** 1 Coordination Center for Assistance Dogs, The Interuniversity Messerli Research Institute of the University of Veterinary Medicine Vienna, Medical University Vienna and University Vienna, 1210 Vienna, Austria; karl.weissenbacher@vetmeduni.ac.at; 2 Karl Landsteiner Research Institute for Neurochemistry, Neuropharmacology, Neurorehabilitation and Pain Treatment Mauer-Amstetten, 3362 Mauer-Amstetten, Austria; lisa.molecular@gmail.com; 3 Dogs and Science, 8142 Uitikon-Waldegg, Switzerland; annika.bremhorst@unibe.ch; 4 Clinical Anesthesiology, Department of Clinical Veterinary Medicine, Vetsuisse Faculty, University of Bern, 3012 Bern, Switzerland

**Keywords:** guide dogs, working dogs, independent evaluation, stress assessment, salivary cortisol, behaviour analysis, task performance, certification protocols, dog welfare, blind handlers

## Abstract

Guide dogs are essential in supporting the autonomy of blind and visually impaired individuals. Austria is the first country to implement a legally mandated, two-stage certification protocol for the official recognition of guide dogs, assessed by an independent authority. The first stage includes two evaluation phases: the dog guides its familiar trainer (Phase 1) and then an unfamiliar blind tester (Phase 2). While Phase 2 ensures an objective assessment of guiding performance, it may also introduce stress due to the unfamiliar handler and separation from the trainer that could impact welfare and behaviour. This study aimed to evaluate whether Phase 2 of the Austrian evaluation protocol elicits higher stress than Phase 1 in guide dogs and, hence, needs to be refined in this regard by comparing physiological and behavioural responses between the two test phases. Fourteen guide dogs were assessed during real evaluations. Salivary cortisol was collected before the evaluation day and at several time points on the evaluation day (before and after each phase). Behavioural responses were coded from video recordings of the first 5 and 15 min of each phase, including stress-related behaviours, task-related performance behaviours, and handler behaviours. Cortisol levels did not differ significantly between the phases. Dogs turned around significantly more often in Phase 2, potentially seeking reassurance, but showed a non-significant trend toward fewer stress-related behaviours. Verbal praise occurred more frequently with the unfamiliar tester. These findings suggest that the current evaluation protocol does not induce substantial physiological or behavioural stress when dogs are guided by an unfamiliar tester, supporting its continued use as a welfare-conscious and objective certification approach that could also potentially serve as a model for other countries.

## Simple Summary

Guide dogs support blind and visually impaired individuals by enabling safe, independent mobility. Austria is the first country to legally mandate that each guide dog be certified by an independent authority. This certification includes a two-phase evaluation: in Phase 1, the dog guides the familiar trainer; in Phase 2, he guides an unfamiliar blind tester. While Phase 2 ensures an objective assessment of guiding performance, it may also place considerable stress on the dog—potentially affecting welfare and performance. This study evaluated whether Phase 2 induces elevated stress in dogs and whether the protocol requires refinement by comparing the dogs’ responses in the two phases. The data was collected during a real guide dog evaluation. We measured salivary cortisol levels before the evaluation day and at several time points on the evaluation day (before and after each phase). We also recorded the dogs’ behaviour and analysed both short-term (first 5 min) and longer-term (15 min) responses in each phase. Human handler behaviours were included as well. Cortisol levels did not differ significantly between the phases. Dogs turned around more frequently when guiding the unfamiliar tester, possibly seeking reassurance, but showed a trend toward fewer stress-related behaviours. The tester gave more verbal praise, which may have helped reduce stress. Overall, the evaluation protocol appears not to cause undue stress and supports welfare-sensitive guide dog certification.

## 1. Introduction

Guide dogs play an essential role in assisting blind or visually impaired individuals by enhancing their mobility, independence, and social inclusion [1,2]. Beyond their functional role in navigation, guide dogs offer emotional support and companionship and are often considered integral members of the family [3,4]. To ensure that guide dogs are both behaviourally capable and emotionally suited to their demanding role, certification procedures are widely used. Yet, little scientific attention has been paid to the potential stress such procedures may cause for the dogs themselves. Certification procedures with familiar and unfamiliar handlers confirm the ability of working dogs to perform a skill, regardless of who provides the cues. The certification process is not merely a formality but a safeguard ensuring that only dogs with the appropriate behavioural and emotional resilience are matched with individuals who depend on them daily. Research suggests that the perceived compatibility between guide dogs and visually impaired owners is marked by an overall high level of satisfaction [5], but nonetheless more than a third of guide dogs are returned to training organisations before retirement [6].

Despite their critical contributions, the regulation and certification processes for guide dogs vary significantly across European countries. This lack of consistency in standards—particularly regarding access rights and certification criteria—poses challenges for guide dog handlers, complicates the independence of the human–dog dyad [7], and raises concerns about the absence of unified, independent statutory inspections to evaluate the dogs’ capabilities and welfare.

As the demand for guide dogs increases, so does the need for standardised evaluation protocols. Currently, most inspection and qualification criteria are defined by individual guide dog training centres and associations, which can lead to variability and potential bias. By contrast, independent testing offers a more objective and transparent framework for assessing a dog’s health, task performance, environmental safety, and suitability for its role. Such evaluations increase the likelihood that guide dogs meet the highest quality standards, fostering trust in the certification process and safeguarding both the welfare of the dogs and the needs of their handlers.

Austria stands out as a pioneering country in the certification and welfare of guide dogs. In 1999, it became the first country to enact a national law (Section 39a Federal Disability Act) that formally defines the requirements, legal status, and qualifications for guide dogs. This legislation also established an independent audit centre to evaluate assistance dogs, ensuring that certified guide dogs meet rigorous standards of health, behaviour, and training. Since then, over 100 guide dogs have been certified in Austria, with around 30 examinations conducted annually.

The Austrian certification process consists of two main steps. First, a quality evaluation assesses the dog’s individual performance and training while working with the familiar trainer who prepared the dog for his working role and with an unfamiliar blind tester. Given the efforts and costs related to guide dog raising and training, the role of the unfamiliar blind tester in the Austrian protocol is necessary in the current protocol of the certification process to identify successful canine candidates. Only dogs that pass this evaluation can later proceed to the second step: a team assessment conducted after the dog has spent several weeks to months with its future handler, who is blind or visually impaired. This process ensures that both the dog’s capabilities and the human–dog partnership are carefully evaluated—offering a model that balances high performance expectations with a commitment to animal welfare.

The quality evaluation itself is conducted in a naturalistic setting and includes two test phases. In Phase 1, the dog guides his familiar trainer through a public route. In Phase 2, the dog guides an unfamiliar blind tester, with the trainer required to leave the testing environment entirely to eliminate any potential visual, olfactory, or auditory cues. This separation is intended to ensure that the dog’s behaviour is not influenced by the presence of the familiar person, thereby allowing for an objective assessment of the dog’s performance under unfamiliar conditions. This second phase enables an objective assessment by an independent blind or visually impaired evaluator but may also place additional emotional demands on the dog. However, while the unfamiliar handler condition is crucial for evaluating the dog’s ability to perform independently of its trainer, it raises questions about potential stress and its impact on performance.

Efforts within the Austrian certification protocol have been made to minimise stress during the examination process, as stress can compromise both guide dog welfare and the validity of performance assessments. This study aimed to evaluate whether the current two-phase Austrian protocol strikes an appropriate balance between objective evaluation and the emotional well-being of the dogs. To address this, we assessed both physiological and behavioural indicators of stress and performance across the two test phases—guiding a familiar trainer and an unfamiliar blind tester. In addition, we considered the behaviours of the human partners (trainer and tester), as these may influence dog behaviour. To gain a nuanced understanding of both immediate and evolving responses, we analysed the first 5 min and the full 15 min of each test phase separately.

Because dogs cannot verbally express internal states like stress, we must rely on measurable proxies. One widely used physiological proxy is salivary cortisol, widely used in dog welfare research as a non-invasive proxy for HPA axis activation as it has been associated with elevated arousal in dogs, for instance during separation from their handler (as described in [8]). However, the reliability of salivary cortisol in pets and working dogs has recently been called into question. A growing body of evidence highlights substantial intra- and inter-individual variability as well as limited correlation with serum levels, particularly under real-world conditions [8,9]. To strengthen the assessment of canine stress, we combined the physiological measurements with behavioural observations. Behavioural indicators such as lip licking, yawning, or body shaking are commonly used markers of stress and emotional arousal in dogs [10,11,12]. In addition, task-related performance behaviours—such as the ability to follow verbal cues by the handler, collaborate, and guide effectively—provide insight into how dogs cope with the cognitive and emotional demands of the evaluation. The behaviour of the human partner may further moderate the dog’s experience, either buffering or amplifying stress.

This multi-modal, context-sensitive approach aims to provide a comprehensive picture of dogs’ emotional and behavioural responses during certification. We hypothesised that dogs would show lower stress levels—both physiologically (as reflected in salivary cortisol) and behaviourally (as reflected in stress-related behaviours)—as well as more stable task-related performance when guiding their familiar trainer compared with an unfamiliar blind tester.

By systematically comparing physiological and behavioural data across the two phases of the Austrian evaluation, this study provides an evidence-based assessment of whether the current protocol appropriately balances welfare considerations with the need for independent, objective certification. The findings are intended to inform refinements of guide dog assessment procedures and contribute to internationally applicable standards that prioritise both functional performance and animal well-being.

## 2. Materials and Methods

### 2.1. Ethical Considerations

The procedures of this study were approved by the Ethics and Welfare Committee of the University of Veterinary Medicine Vienna, in accordance with GSP guidelines and national legislation (reference number ETK-11/03/2016). Written informed consent was obtained from all dog owners prior to participation.

### 2.2. Subjects

Fourteen guide dogs participated in this study during their real quality evaluation phase of the Austrian certification process for official recognition as guide dogs. The sample included six breeds: 10 Retrievers, 2 Collies, 1 Poodle, and 1 Labrador crossbreed. All dogs were neutered (8 females, 6 males) and ranged in age from 18 to 45 months (mean = 26.3 months, median = 24.5 months; also see Table 1). Each dog was owned by their respective guide dog trainer and had successfully passed comprehensive veterinary health screenings prior to the evaluation, including radiographic assessments of hips, shoulders, and elbows.

### 2.3. Procedures

Data collection was conducted during the formal guide dog evaluation as part of the Austrian certification process, taking place under real-life conditions. Each evaluation involved up to six individuals, including the guide dog trainer, an examination committee (comprising a blind or visually impaired tester, an expert in dog behaviour and training, and an examination chair), a mobility trainer, and the experimenter.

The evaluation protocol started at the Messerli Research Institute, located on the campus of the University of Veterinary Medicine in Vienna, Austria. The evaluation consisted of two test phases:

- Phase 1—Trainer Test: In the first 45 min session, the guide dog trainer, blindfolded to simulate visual impairment, was guided by the dog along a predefined public route, beginning on the campus (including a brief obedience task) and continuing through urban environments.
- Break: Following Phase 1, a break of at least 30 min was provided in a calm, park-like setting to allow the dog to rest, drink water, and gradually become acquainted with the blind tester.
- Phase 2—Tester Test: In the second 45 min session, the dog was tasked with guiding the unfamiliar blind tester along the return route back to campus. However, a different route as in Phase 1 was used. To ensure the independence of the evaluation, the trainer was instructed to leave the scene entirely—out of sight, hearing, and smell range of the dog—unless the dog refused to proceed with the unfamiliar person. In such cases (observed in one dog, Armani), the trainer followed the dog and tester at close distance to provide support without interfering with the test. This phase also included navigating various forms of public transport (e.g., tram, bus, and subway), further reflecting the realistic challenges of everyday life.

### 2.4. Data Collection and Analysis

#### 2.4.1. Physiological Measure: Salivary Cortisol

Saliva samples were collected to measure cortisol levels at the Institute of Medical Biochemistry at the University of Veterinary Medicine in Vienna using a validated enzyme immunoassay [13] as used in several previous studies [14,15,16]. All saliva samples (except two samples) were taken by the guide dog trainer in less than 4 min to prevent the procedure causing any stress to the dog [17]. The guide dog trainers were trained to collect saliva samples using standardised protocols, including detailed written instructions and demonstration images. To minimise contamination, the trainers wore gloves and used cotton swabs (Salivette^®^, Sarstedt, Biedermannsdorf, Austria), which were gently rubbed inside the dog’s cheek for approximately 60 s. If insufficient saliva was produced, the trainers used olfactory stimulation (e.g., holding a food reward) to encourage salivation. The samples were stored in iceboxes during exams and later frozen at −20 °C for further analysis.

##### Sampling Schedule

**Figure 1:**
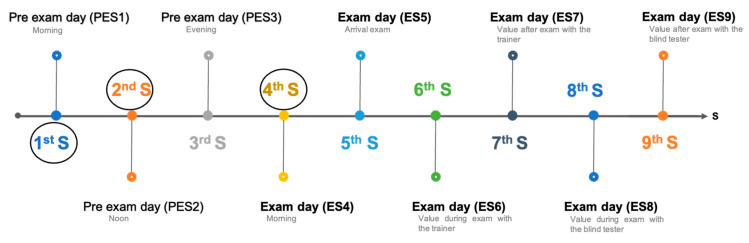
Description of saliva sampling on pre-evaluation day and evaluation day. S = sample, circles = baseline derived from calculating the average of the measured salivary cortisol levels from the sampling of PES1, PES2, and PES4 (design by PresentationGO.com and adjusted by Viola Färber-Morak).

Saliva samples were collected at multiple predefined sampling time points on the day before the evaluation (pre-evaluation day) and on the day of the evaluation (see Figure 1).

Pre-evaluation day sampling time points (PES1–PES3):

- PES1: Morning, immediately after waking, before feeding or exercise.
- PES2: Noon, approximately six hours after PES1.
- PES3: Evening, approximately six hours after PES2.

Evaluation day sampling time points (ES4–ES9):

- ES4: Morning, immediately after waking, before feeding or exercise.
- ES5: Start of Phase 1—Trainer Test.
- ES6: End of Phase 1—Trainer Test.
- ES7: Start of Phase 2—Tester Test.
- ES8: End of Phase 2—Tester Test.
- ES9: Approximately 30 min after ES8.

To align with the approach of Glenk and co-authors [18], the sampling time points PES1, PES2, and ES4 were selected to represent baseline cortisol levels in the home environment across different times of day. Due to the potential influence of circadian rhythms on cortisol secretion, the evening sample (PES3) was excluded from the baseline calculation to avoid potential confounding effects. Instead, a baseline value was calculated by averaging the salivary cortisol concentrations from PES1, PES2, and ES4.

The sampling schedule was designed to capture the time course of cortisol in relation to the two test phases of the guide dog evaluation (approximately 45 min each). Given that cortisol typically peaks 20–30 min after a stressful event [19], the timing of samples was selected to detect potential physiological responses relevant to the study aims.

##### Analysis

Descriptive statistics (means and standard deviations) were calculated for six salivary cortisol measures: the computed baseline (average of PES1, PES2, and ES4) and the five individual post-baseline time points (ES5, ES6, ES7, ES8, and ES9). The Kolmogorov–Smirnov test was used to assess the normality of the data, and log transformations were applied where necessary to improve normality. To evaluate differences in cortisol concentrations across the six measurement points, a repeated measures ANOVA was conducted using the log-transformed values. Corrections for potential violations of sphericity (Greenhouse–Geisser and Huynh–Feldt) were applied to ensure the robustness of the analysis. Post hoc pairwise comparisons were conducted with adjustments for multiple testing to identify specific differences between time points. To further investigate potential time-related effects, multivariate tests were conducted to assess the combined effects of time on the dependent variables across all sampling points.

#### 2.4.2. Behavioural Measures

##### Sampling

The behaviour of the dogs during both test phases (Trainer and Tester) was recorded using a GoPro Hero 4 camera (San Mateo, CA 94402, U.S.), mounted on the upper body of the person being guided (either the trainer or the blind tester). Video recordings were subsequently analysed using Solomon Coder Beta 17.03.22 (Copyright András Péter).

Video coding began at the moment the human handler—either the trainer or the tester—issued the first guiding cue to the dog, initiating movement. For each phase, the first 15 min of active guiding were coded to capture both immediate and sustained behavioural responses.

##### Behavioural Variables

We analysed three categories of behaviours: stress-related behaviours, task-related performance behaviours, and handler behaviours. All behaviours were coded in terms of their frequency of occurrence during the observation periods.

Stress-related behaviours: Six stress-related behaviours were selected based on the previous literature [10,11,12,17,18,20,21,22,23]. These behaviours included lip licking, shaking, smacking, scratching, stretching, and yawning (see Table 2). To quantify overall behavioural stress responses, a Standardised Stress Score (SSScore) was created from these variables. Due to its very low frequency, stretching was excluded from the final score.

To compute the SSScore, the frequency of each behaviour was first standardised using z-scores. Standardisation involved subtracting the mean frequency of a behaviour from the individual scores and dividing by the standard deviation. This approach adjusted for variance among behaviours and normalised the contribution of each behaviour, ensuring they were comparable on the same scale. The standardised z-scores were then combined to produce the SSScore, preventing any single behaviour from disproportionately influencing the measure due to differences in scale or frequency distribution. The final SSScore was calculated as a mean index of the five standardised stress behaviours (lip licking, shaking, smacking, scratching, and yawning).

Task-related performance behaviours: These behaviours reflect key aspects of the dog’s task performance and included the variables Refuse signal and Turn around (Table 2). They were chosen for their relevance to the dog’s collaboration and guidance abilities, and because they were reliably identifiable from the video perspective. Behaviour definitions were discussed and agreed upon in advance to ensure consistent coding.

Handler behaviours: Two variables were coded to assess human behaviour during the test: Praise (verbal reinforcement) and Treat (food-based reward; see Table 2). These variables provided context for the dog’s behavioural responses and were considered potential moderating factors in stress or performance.

##### Statistical Analysis

Reliability analysis: To ensure the reliability of the behavioural coding, intraclass correlation coefficients (ICCs) were calculated for each coded behaviour. This allowed us to assess the consistency of ratings provided by two independent observers (the second observer coded a subsample of videos (from four of the subjects, which were randomly selected) and confirm the robustness of the behavioural data.

Modelling behavioural data: To gain a nuanced understanding of both the immediate and evolving behavioural responses, we analysed the first 5 and 15 min of each test phase (Trainer and Tester) separately. These two timeframes allowed us to distinguish between the initial reactions to the testing situation and behavioural patterns that emerged or changed over time.

We used linear mixed models to investigate the effects of key predictors on the behavioural responses of the dogs. The dependent variables included the stress-related behaviours (SSScore), the task-related performance behaviours (Refuse signal and Turn around), and the handler behaviours (Praise and Treat). The fixed effects included test phase (Phase 1—Trainer; Phase 2—Tester), dog sex (male or female), and age (in months). To account for repeated measures and inter-individual variability, each dog was included as a random effect in the models. Where necessary, transformations were applied to the behavioural data to meet the assumptions of normality required for linear modelling.

## 3. Results

### 3.1. Physiological Parameter: Salivary Cortisol

#### 3.1.1. Preliminary Analyses

##### Descriptive Statistics

The raw cortisol concentrations ranged from 1.879 ng/mL (SD = 1.712) at ES8 to 2.736 ng/mL (SD = 1.846) for the baseline. To meet assumptions of normality, logarithmic transformations were applied. After logarithmic transformation, the means ranged from 0.0043 ng/mL (SD = 0.613) at ES7 to 0.2452 (SD = 0.341) at ES9. Data completeness was 100% for all time points except ES6 (one sample missing for dog Maya).

##### Normality Testing

The Kolmogorov–Smirnov tests indicated non-normality for all variables except the baseline (*p* = 0.200). Significant deviations were found for ES5, ES6, ES7, ES8, and ES9 (*p* < 0.05). Log transformations improved normality across most variables (*p* > 0.05), and the log-transformed data were therefore used for further analyses, despite a slight reduction in normality for the baseline (*p* = 0.008).

#### 3.1.2. Inferential Analyses

##### Repeated Measures ANOVA

To assess the changes in cortisol over time, a repeated measures ANOVA was conducted using the six log-transformed cortisol values (baseline, ES5–ES9). The analysis revealed no significant overall effect of time (F(5,60) = 0.811, *p* = 0.546). This result remained non-significant when applying corrections for sphericity violations (Greenhouse–Geisser: *p* = 0.494; Huynh–Feldt: *p* = 0.523).

##### Pairwise Comparisons Between Time Points

**Figure 2:**
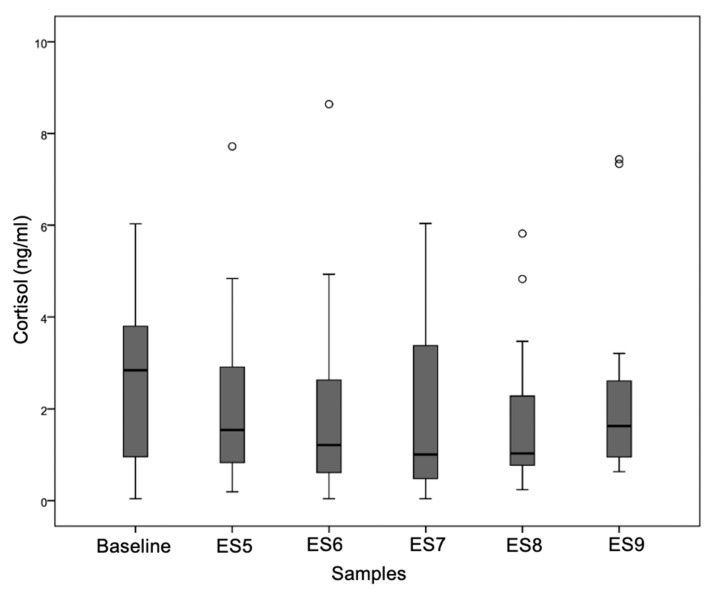
Box plot illustrating the time course of salivary cortisol concentrations (ng/mL) across the six sampling points. Cortisol levels declined from baseline (mean of PES1, PES2, and ES4; n = 14 individuals) through to ES5 (start of evaluation with Trainer; n = 14; ° = outliers), ES6 (end of evaluation with Trainer; n = 13), and reached their lowest point at ES7 (start of evaluation with Tester; n = 14). Levels then increased again during ES8 (end of evaluation with Tester; n = 14) and ES9 (post evaluation; n = 14). Although these changes followed a clear descriptive pattern, statistical analysis revealed no significant differences in cortisol concentrations across time points.

Pairwise comparisons between the six log-transformed cortisol values showed no statistically significant differences (all *p* > 0.05; Figure 2). The largest mean difference was observed between the baseline and ES7 (−0.272, *p* = 0.229), while the comparison between ES7 and ES9 yielded the lowest *p*-value (mean difference = −0.250, *p* = 0.112).

##### Cortisol Profile

Although statistical differences were absent, the cortisol profile (Figure 2, see Supplementary Materials) showed a descriptive pattern: levels appeared to decrease during the test with the familiar trainer and rise again during and after the test with the unfamiliar blind tester. These trends suggest individual variability but do not indicate a consistent or significant physiological stress response to the test conditions.

### 3.2. Behavioural Parameters

#### 3.2.1. Reliability Analysis

Overall, the ICC values indicated excellent inter-rater reliability across most behavioural variables. Perfect agreement (ICC = 1.00) was observed for several behaviours, including shaking, scratching, and yawning. While some ICC values could not be computed due to uniform ratings (resulting in undefined variance), all available ICCs exceeded acceptable thresholds for reliability. Table 3 provides a detailed overview of the ICCs, confidence intervals, F-test values, and significance levels for each behaviour.

#### 3.2.2. Behavioural Data Analysis: Early Response (First 5 min)

Stress-related behaviours: The Standardised Stress Score (SSScore), which quantifies stress-related behaviours, did not differ significantly between the two test phases (Trainer vs. Tester). Similarly, no significant effects were found for sex or age (see Table 4 and Table 6).

Task-related performance behaviours: The frequency of the log-transformed “Turn around” behaviour was significantly higher in Phase 2 (Tester) compared with Phase 1 (Trainer; *p* = 0.0251; see Supplementary Materials), suggesting that dogs were more likely to look back when guiding an unfamiliar person. A marginally significant effect of sex was observed (*p* = 0.0561), with male dogs tending to perform this behaviour more frequently than females. Age did not significantly influence this variable. In contrast, the frequency of the log-transformed “Refuse signal” behaviour was not significantly affected by test phase, sex, or age (Table 4 and Table 6).

Handler behaviours: Neither of the two handler-related behaviours—“Praise” and the log-transformed “Treat”—showed significant effects of test phase, sex, or age (Table 4 and Table 6; see Supplementary Materials).

#### 3.2.3. Behavioural Data Analysis: Extended Response (First 15 min)

Stress-related behaviours: The SSScore showed a marginal effect of test phase (*p* = 0.0576), with dogs displaying slightly lower stress scores in Phase 2 (Tester) compared with Phase 1 (Trainer). No significant effects of sex or age were observed (see Table 5 and Table 6).

Task-based performance behaviours: The frequency of the “Turn around” behaviour was significantly higher in Phase 2 (Tester) than in Phase 1 (Trainer; *p* = 0.007), indicating that dogs were more likely to look back when guiding an unfamiliar person. A significant effect of sex was also found (*p* = 0.024), with male dogs performing this behaviour more frequently than females. Age had no significant effect. For “Refuse signal”, no significant influence of test phase, sex, or age was detected, although descriptively, the behaviour occurred more often in Phase 2 (Tester; Table 5 and Table 6; see Supplementary Materials).

Handler behaviours: The behaviour “Praise” occurred significantly more frequently in Phase 2 (Tester) than in Phase 1 (Trainer; *p* = 0.0115), whereas “Treat” showed a marginally significant decrease in Phase 2 compared with Phase 1 (*p* = 0.0569). Neither sex nor age significantly influenced these variables (Table 5 and Table 6; see Supplementary Materials).

## 4. Discussion

In the present study, we found no evidence that guiding an unfamiliar blind tester induced higher stress in dogs compared with guiding their familiar trainer. Cortisol levels remained stable across phases, and behavioural stress indicators (SSScore) did not differ significantly. However, dogs turned around significantly more often when guiding the unfamiliar tester—potentially seeking reassurance. The tester also gave significantly more verbal praise, which may have helped to buffer stress. Overall, the evaluation protocol did not appear to elicit substantial physiological or behavioural stress, supporting its continued use as a welfare-conscious certification approach.

Although the descriptive patterns of the salivary cortisol measures suggested a decrease during the test with the familiar trainer and appeared to increase again during the test with the blind tester, these trends did not reach statistical significance. Studies that particularly focused on canine stress reported that cortisol increases beyond baseline thresholds when the dog is confronted with arousing stimuli [24,25]. Moreover, human interaction style and personality have been shown to affect their dogs’ cortisol secretion during sequences of interaction [26,27]. The fact that cortisol concentrations assessed at the end of the evaluation (following the phase of interaction with the unfamiliar person and reunion with their familiar trainer) were similar to home baseline values do not point to any substantial strain caused by the examination protocol or change in handler. Previously reported elevations in cortisol levels among guide dogs, as compared to companion dogs, suggest a sustained activation of the HPA axis that is potentially linked to the unique demands of their working role [28].

However, the interpretation of salivary cortisol must be approached with caution. Meta-analyses and recent studies have highlighted substantial intra- and inter-individual variability [8], as well as weak correlations with serum cortisol in real-world settings [9]. Also the cortisol data gathered in this study point to a high variability. These findings raise questions about the validity of salivary cortisol as a standalone stress marker, particularly in applied working dog contexts. Importantly, this reinforces the need to complement hormonal measures with behavioural data, which may offer greater ecological validity.

The Standardised Stress Score, aggregating multiple behavioural indicators of stress (e.g., lip licking, yawning, and shaking), did not differ significantly between the two test phases during the early (5 min) observation. However, during the extended (15 min) phase, the SSScore tended (*p* = 0.058) to be lower when dogs guided the unfamiliar blind tester compared to their familiar trainer—a finding that contrasts with our initial hypothesis. One possible explanation for this unexpected pattern could lie in the behaviour of the handler. The blind tester provided significantly more verbal praise than the trainer during the evaluation. Verbal praise has been shown to act as a form of social support and stress buffering in dogs [29,30], which may have moderated the dogs’ arousal levels during this phase. Interestingly, while the trainer tended to offer more food treats, this difference did not reach significance.

Another possible explanation for the patterns we found in the present study could lie in the phenomenon of emotional contagion—the transfer of emotional states between individuals. Emotional contagion between dogs and humans is well documented in the literature [31,32,33]. For humans, examinations are perceived as a potent source of stress and fear, which consequently affects cortisol secretion and cognitive performance [34]. One moderating factor in the human–dog emotional contagion is the duration of dog ownership [35], which ultimately is connected to the intensity of the relationship. In fact, recent data indicate that dogs exhibit less empathetic behaviour to strangers in unfamiliar environments [36] but in owner–dog dyads, empathetic traits predispose dogs to be influenced by their owners’ anxiety [37]. Given that the dogs had built an intense relationship with their trainer but not with the blind tester, it could be possible that they were, therefore, more likely to be affected by the stress load of their familiar handler in this examination situation which could result in the observed behaviour effect. Similarly, the blind tester was also likely less concerned about the outcome of the certification and, therefore, less emotionally involved.

However, this interpretation remains speculative, as we did not assess the physiological or behavioural indicators of stress in the human handlers. Yet, previous research has shown that handlers’ stress levels can influence working dog behaviour [38,39], and even in pet dogs, long-term cohabitation with a stressed owner has been associated with elevated canine cortisol levels [40]. Future studies should therefore include measures of human stress (e.g., heart rate variability and self-report questionnaires) to fully understand the dyadic dynamics at play during certification.

The frequency of the “Turn around” behaviour—which could be interpreted as seeking visual contact or reassurance—was significantly higher in Phase 2 than in Phase 1 in both the early and extended observation periods. Data by Wanser & Udell [41] suggest that insecurely attached therapy dogs tended to gaze more often at their handlers while working. Our finding suggests that the dogs may have experienced some uncertainty or discomfort when guiding the unfamiliar person, potentially looking back to locate their trainer or reassess the situation. This behaviour was also more frequent in male dogs, thereby adding to the existing body of sex-based differences in canine social behaviour [42]. Sex differences in canine behaviour have been widely reported including aspects like aggression, fearfulness, playfulness, gazing, preferred paw use, and sociability with strangers [42]. The results of the review by Scandurra and collaborators [42] suggest that in general, females scored higher on sociability and cooperation with strangers. In line with these findings, the female dogs in our study possibly were more willing to cooperate with an unfamiliar person and because of that they may have needed less visual reassurance.

Despite these subtle behavioural differences, the dogs generally continued to perform reliably across both phases when guiding their familiar trainer and unfamiliar tester. There were no significant differences in the frequency of “Refuse signal” behaviours—an important task-related performance measure—between phases, suggesting that the dogs were able to execute cues regardless of the handler’s familiarity. This also supports the notion that guide dogs are capable of transferring their training to new contexts and individuals.

Our results also highlight the importance of human behaviour during evaluations. While task performance is often seen as a measure of the dog’s capability alone, our findings indicate that the interaction style of the handler may influence the dog’s emotional state. Increased praise in Phase 2 may have mitigated stress, whereas fewer treats may reflect different reinforcement strategies such as verbal praise, eye contact, or body language. These observations support the calls for including handler behaviour as a variable in future certification research.

The trends observed in the current study warrant further investigation to validate the robustness of these findings. Such insights could inform the further refinement of international certification standards, ensuring that independent evaluations, like the Austrian protocol, continue to prioritise both welfare and functional performance. Our findings also underscore the importance of evaluating stress responses in a nuanced manner and highlight the need for further research to account for the handler’s influence on the dog’s stress levels. The nuanced behavioural trends—such as increased “Turn around” behaviour and differences in human interaction styles—highlight the complexity of assessing stress in working dogs. Future studies should aim to replicate these findings with larger sample sizes and expand the range of physiological indicators by including additional biomarkers such as heart rate variability [43] or salivary immunological markers [44]. Importantly, human-related variables, such as the stress levels and interaction styles of trainers and testers, should also be systematically measured.

Of note, the present findings rely on the Austrian protocol and, therefore, cannot be generalised to the certification standards in other countries. However, some preliminary insights were gained on dog welfare indicators and performance during guide dog certification, which to date, is a relatively unexplored area of research.

## 5. Conclusions

To conclude, our hypothesis that handling by an unfamiliar tester universally induces heightened stress and so guide dogs would exhibit higher stress and reduced performance when guiding an unfamiliar tester was not supported by our data. Instead, the findings suggest that dogs seem to adapt well to the unfamiliar context and do not seem to experience substantial stress during the evaluation. The continued use of an unfamiliar tester thus appears justifiable as a means of ensuring objectivity, especially as it allows performance to be assessed independently of the dog’s established relationship with the trainer. Based on our findings, the current Austrian protocol appears to strike an appropriate balance between maintaining dog welfare and achieving objective assessments. As such, it may serve as a potential model for other countries or guide dog organisations seeking to develop or refine certification procedures that prioritise both transparency and animal well-being.

## Figures and Tables

**Table 1:** Dog name, breed, age, sex, availability of cortisol and behavioural data, and demographic characteristics of trainers and testers. ok = data available, ✕ = data not available.

Name	Breed	Age Months	Sex	Dog Cortisol Data	Dog Behavioural Data	Trainer Sex	Tester Sex
Elisa -Pixie	Collie (long hair)	30	f	ok	ok	f	f
Tasha	Labrador Retriever	23	f	ok	ok	m	m
Mira	Labrador Retriever	26	f	ok	ok	m	f
Darla	Labrador Retriever	25	f	ok	ok	m	f
Sam	Labrador-Hovawarth mixed breed	45	m	ok	ok	m	m
Hadis	Curly Coated Retriever	32	m	ok	ok	f	m
Harmony	Curly Coated Retriever	21	f	ok	ok	f	m
Darell	Labrador Retriever	26	m	ok	ok	m	m
Maya	Labrador Retriever	18	f	✕	ok	m	m
Pablo	Labrador Retriever	24	m	ok	ok	m	m
Coco	Flat Coated Retriever	22	f	ok	ok	f	f
Armani	Poodle	23	m	ok	ok	f	f
Colleen	Collie (long hair)	29	f	ok	✕	f	✕
Sultan	Golden Retriever	24	m	ok	✕	m	✕

**Table 2:** Ethogram with descriptions of coded dog (stress- and task-related performance behaviours) and handler behaviours. Stress-related behaviours marked with an asterisk (*) were included in the calculation of the Standardised Stress Score (SSScore).

Category	Behaviour	Definition
Stress-related behaviours (dog)	Lip licking *	Tongue is moving from the front part of the mouth sideways to the upper lip
	Smacking *	Dog opens and closes mouth immediately
	Yawning *	Dog opens the mouth widely to the fullest extent while moving the ears sideways
	Shaking *	Moving mostly the whole part of the body from one side to the other starting from the head caudally
	Scratching *	Scratching a body part with one of the hind limbs
	Stretching	Putting the front of the body down while lengthening the front limbs and/or the hind limbs while holding one limb after each other in the air
Task-related performance behaviours (dog)	Refuse signal	Handler gives a cue and dog does not respond to the cue
	Turn around	Dog turns head more than 90 degrees to the left or right side and looks back over shoulder
Handler behaviours (human)	Treat	Handler is delivering food reward to the dog
	Praise	Handler is praising dog verbally

**Table 3:** This table presents the single score intraclass correlation coefficients (ICC) for the various behaviours rated by the two observers. The ICC values assessed the consistency between the raters, with the model focusing on consistency. The table includes the ICC values, 95% confidence intervals, F-test statistics, and *p*-values for each behaviour.

Behaviour	ICC	95% CI	F-Test	*p*-Value
Lip licking	0.992	0.963–0.998	F(7,8) = 240	<0.001
Shaking	1.00	Not computable		
Smacking	0.832	0.413–0.963	F(7,8) = 10.9	0.002
Scratching	1.00	Not computable		
Yawning	1.00	Not computable		
Turn around	0.823	0.389–0.961	F(7,8) = 10.3	0.002
Refuse signal	0.936	0.741–0.987	F(7,8) = 30.4	<0.001
Praise	0.991	0.962–0.998	F(7,8) = 231	<0.001
Treat	0.963	0.841–0.992	F(7,8) = 52.6	<0.001

**Table 4:** Results of linear mixed models for behavioural responses during the first 5 min of each test phase. This table summarises the effects of test phase (1—Trainer, 2—Tester), sex (male/female), and age (in months) on: (1) stress-related behaviours (Standardised Stress Score, SSScore), (2) task-related performance behaviours (Refuse signal, Turn around), (3) handler behaviours (Praise, Treat). The table includes estimates, standard errors (SE), degrees of freedom (Df), t-values, *p*-values, and the direction of significant effects where applicable (also for marginal effects).

Parameter	B	SE	Df	t	*p*
(1) Stress-related behaviours: SSScore					
Phase 2 (Tester)	−0.191515	0.20	20.00	−0.944	0.356
Sex (Male)	0.260870	0.23	20.00	1.118	0.277
Age (Months)	−0.007416	0.02	20.00	−0.433	0.670
(2) Task-related performance behaviours					
Turn around					
Phase 2 (Tester)	0.955402	0.37	10.999975	2.592	0.025
Sex (Male)	0.956772	0.44	8.999980	2.191	0.056
Age (Months)	0.002562	0.03	8.999980	0.080	0.938
Refuse signal					
Phase 2 (Tester)	0.59747	0.41	10.98185	1.451	0.175
Sex (Male)	−0.01532	0.47	8.98190	−0.032	0.975
Age (Months)	0.01973	0.03	8.98191	0.567	0.585
(3) Handler behaviours					
Praise					
Phase 2 (Tester)	3.00000	2.00	11.00000	1.497	0.163
Sex (Male)	−3.03543	2.69	9.00000	−1.130	0.288
Age (Months)	−0.04782	0.20	9.00000	−0.242	0.814
Treat					
Phase 2 (Tester)	0.10033	0.27	20.00000	0.373	0.713
Sex (Male)	−0.27617	0.31	20.00000	−0.893	0.383
Age (Months)	−0.00382	0.02	20.00000	−0.168	0.868

**Table 5:** Results of linear mixed models for behavioural responses during the first 15 min of each test phase. This table summarises the effects of test phase (1—Trainer, 2—Tester), sex (male/female), and age (in months) on: (1) stress-related behaviours (Standardised Stress Score, SSScore), (2) task-related performance behaviours (Refuse signal, Turn around), (3) handler behaviours (Praise, Treat). The table includes estimates, standard errors (SE), degrees of freedom (Df), t-values, *p*-values, and the direction of significant effects where applicable (also for marginal effects).

Parameter	B	SE	Df	t	*p*
(1) Stress-related behaviours: SSScore					
Test Phase (2)	−0.40065	0.19	11.00	−2.120	0.058
Sex (Male)	0.38739	0.31	9.00	1.259	0.240
Age (Months)	−0.02424	0.02	9.00	−1.072	0.312
(2) Task-related performance behaviours					
Turn around					
Test Phase (2)	9.4167	3.14	20.00	3.003	0.007
Sex (Male)	8.8142	3.61	20.00	2.443	0.024
Age (Months)	−0.1955	0.27	20.00	−0.738	0.469
Refuse signal					
Test Phase (2)	6.83333	3.92	20.00	1.742	0.097
Sex (Male)	0.70524	4.51	20.00	0.156	0.877
Age (Months)	0.06807	0.33	20.00	0.205	0.839
(3) Handler behaviours					
Praise					
Test Phase (2)	14.3333	4.73	11.0000	3.028	0.012
Sex (Male)	−9.5996	8.09	9.0000	−1.186	0.266
Age (Months)	−0.1023	0.59	9.0000	−0.172	0.867
Treat					
Test Phase (2)	−0.529676	0.25	10.999961	−2.127	0.057
Sex (Male)	−0.383759	0.32	8.999995	−1.183	0.267
Age (Months)	0.004237	0.02	8.999995	0.178	0.863

**Table 6:** Summary of key results from the physiological and behavioural analyses. This table provides an overview of the main effects of test phase (1 = Trainer; 2 = Tester) on cortisol levels and behavioural variables, based on both early (first 5 min) and extended (first 15 min) observation periods. An asterisk (*) indicates a statistically significant effect (*p* < 0.05) of the test phase.

Category	Variable	Early Period: 5 min	Extended Period: 15 min
Physiological analysis	Cortisol	Phase 1 = Phase 2	
Behavioural analysis			
Stress-related behaviours	SSScore	Phase 1 = Phase 2	Phase 1 = Phase 2
Task-related performance behaviours	Turn around	Phase 1 < Phase 2 *	Phase 1 < Phase 2 *
	Refuse signal	Phase 1 = Phase 2	Phase 1 = Phase 2
Handler behaviours	Praise	Phase 1 = Phase 2	Phase 1 < Phase 2 *
	Treat	Phase 1 = Phase 2	Phase 1 = Phase 2

## Data Availability

The analysed data is provided as Supplementary Materials.

## Supplementary Materials

The following supporting information can be downloaded at: https://www.mdpi.com/article/10.3390/ani15131896/s1, Excel File S1: frequencies_behavioural_data_15min; Excel File S2: frequencies_behavioural_data_first_5min; Excel File S3: guide dogs_cortisol_data.
